# Influence of Biological Therapeutics, Cytokines, and Disease Activity on Depression in Rheumatoid Arthritis

**DOI:** 10.1155/2018/5954897

**Published:** 2018-07-17

**Authors:** Margarida Figueiredo-Braga, Caleb Cornaby, Alice Cortez, Miguel Bernardes, Georgina Terroso, Marta Figueiredo, Cristina Dos Santos Mesquita, Lúcia Costa, Brian D. Poole

**Affiliations:** ^1^Medical Psychology Unit, Department of Clinical Neurosciences and Mental Health, Faculty of Medicine, University of Porto, Porto, Portugal; ^2^I3S Instituto de Investigação e Inovação em Saúde, Porto, Portugal; ^3^Department of Microbiology and Molecular Biology, Brigham Young University, Provo, UT, USA; ^4^Laboratorio Nobre, Faculty of Medicine, University of Porto, Porto, Portugal; ^5^Rheumatology Department, Hospital of São João EPE, Porto, Portugal; ^6^Departamento de Psicologia Aplicada (DPA), Universidade do Minho, Braga, Minho, Portugal

## Abstract

**Purpose:**

Rheumatoid arthritis (RA) is an often debilitating autoinflammatory disease. Patients with rheumatoid arthritis are often troubled by co-occurring depression or other psychological manifestations. RA patients have a variety of treatment options available, including biologicals that inhibit cytokines or immune cells. If these cytokines influence the psychological symptoms, then the use of cytokine inhibitors should modulate these symptoms.

**Methods:**

A cohort of 209 individuals was recruited. This group included 82 RA patients, 22 healthy subjects, 32 depressed control subjects, and 73 subjects with systemic lupus erythematosus. Of the RA patients, 51% were on a biological therapeutic. ELISA was used to measure cytokine levels. A variety of psychological assessments were used to evaluate depression, anxiety, sleep, fatigue, and relationship status. Clinical values were obtained from medical records.

**Results:**

IL-10 concentration was associated with depressive symptoms in the RA patients, healthy controls, and the lupus patients. In the patients with primary depression, depressive symptoms were associated with IL-6 and TNF-alpha. In RA patients, Tocilizumab use was associated with decreased depressive symptoms. 14 RA patients who were not using biologicals began using them by a one-month follow-up. In these patients, there was no significant change to any value except for fatigue.

**Conclusions:**

A variety of both biological and social factors influences depressive symptoms in RA. IL-10 and IL-6 are likely to be involved, since IL-10 concentration was associated with depression and Tocilizumab decreased depressive symptoms in the RA patients. The roles of these cytokines are different in RA and lupus, as high IL-10 in RA is associated with increased depressive symptoms, but high IL-10 in the lupus patients is associated with decreased depression. IL-6 was also associated with depressive symptoms in the patients with primary depression. These results strongly indicate that disease activity, including cytokine levels, has a strong impact on depressive symptoms.

## 1. Background

Rheumatoid arthritis (RA) is a destructive autoinflammatory arthritis characterized by the overproduction of certain proinflammatory cytokines. The disease is destructive in nature, leading to pain, stiffness, bone resorption, and systemic inflammation. Treatment with monoclonal antibodies or antibody derivatives (biologicals) that block specific cytokines has been very successful. Many of these biologicals prevent bone destruction and have a substantial impact on disease activity [1]. Biologicals are also useful in understanding the mechanisms of rheumatoid arthritis, since they block the activity of specific cytokines.

Patients with rheumatoid arthritis have a higher prevalence of depression and anxiety than the general population [2]. Part of this increased prevalence may be due to the toll that chronic, painful disease takes on the quality of life. Another aspect may be the heightened levels of inflammatory cytokines found in rheumatoid arthritis. Several of these cytokines have been linked to depression, such as TNF-alpha [3–6] and IL-6 [7]. IL-10 is often negatively correlated with depression [8, 9]. Depression may influence cytokine activity, as patients with more depressive symptoms are less responsive to biological antirheumatic therapies [10].

In order to investigate the role of cytokines in depression associated with rheumatoid arthritis, we studied the relationship between depression, clinical markers, and serum cytokine levels in rheumatoid arthritis patients, patients being treated for primary depression, healthy controls, and lupus patients. We further hypothesized that cytokine inhibition via biological agents would have an impact on depressive symptoms. To test this hypothesis, a subcohort of rheumatoid arthritis patients being treated with biological therapeutics to inhibit specific cytokines was examined, to determine the effect of cytokine inhibition on depression.

## 2. Materials and Methods

A cohort of 209 individuals was recruited for this study. This group included 82 RA patients, 22 healthy control subjects, 32 subjects with primary depression, and 73 subjects with systemic lupus erythematosus as a control group for autoimmune arthritis (Table 1). Diagnosis and stage of disease activity were established according to the American College of Rheumatology Classification Criteria (ACR) [11], by a rheumatologist. All RA patients attended routine visits at the hospital and completed regular clinic and laboratory assessments. Patients with depressive disorder were diagnosed by a psychiatrist working in a psychiatric private clinic according to the Diagnostic and Statistical Manual of Mental Disorders (DSM-IV-TR). Female healthy controls were recruited using a snowball approach and included in the sample. Patient recruitment was made during a consultation. Once accepted, the researcher further informed the participants in a separate room of the study aims and procedures and all who agreed to participate received written and verbal information about the study and signed an informed consent form and were invited to perform a peripheral blood collection. A telephone call was scheduled to perform the psychosocial evaluation, and recruited patients and controls were subsequently interviewed by phone by a psychologist. The literature corroborates phone interviews as valid and precise tools for psychological data collection. [12]. Laboratory and clinical evaluations were obtained for the RA and lupus patients through clinical records. Lab tests included leukocytes (10^9^/L), lymphocytes (percentage), platelets (10^9^/L), erythrocyte sedimentation rate (mm/h), anti-dsDNA antibody titer (IU/mL), and C-reactive protein level (mg/dL). Disease activity was assessed by both the patient and the doctor, and the DAS28 was used for overall evaluation. Physical activity, smoking, and alcohol consumption were also recorded. Physical activity was assessed based on involvement in sporting activities.

The study was submitted and approved by the Ethical Committee of the São João Hospital IRB (EPE) in accordance with the Declaration of Helsinki. The nature and the purpose of the study were explained to all participants who signed the informed consent form before they entered the study.

### 2.1. Psychosocial and Clinical Evaluation

Sociodemographic characteristics were included in the questionnaire. Education level was given as years of school. Age and marital status were listed. Socioeconomic class evaluation was included using employment status and years of education as proxies. Psychological evaluations were performed using several standardized instruments.

#### 2.1.1. Fatigue Severity Scale (FSS)

The short form of the FSS was used for this study. This form allows the participant to report their perceived level of fatigue [13]. The Portuguese version includes nine items. This instrument has been found to be valid and useful for studies involving patients with systemic lupus erythematosus [14].

The Cronbach *α* is 0.89 for the FSS, and the test-retest reliability is 0.84, which indicates good psychometric properties. The mean of all scored items is used to generate a total score, with higher scores indicating a higher severity of fatigue. Clinical fatigue is defined by a FSS score > 3. The scale has is sensitive to change, and interviews conducted via telephone can use it reliably. The FSS has also been shown to be reliable across different patient populations [15].

#### 2.1.2. Hospital Anxiety and Depression Scale (HADS)

The Hospital Anxiety and Depression Scale (HADS) measures anxiety and depression, especially in people with physical illness [16]. The scale has a Cronbach alpha coefficient of 0.94, indicating good psychometric properties [17]. The HADS is subdivided into two subscales of 7 items. One subscale measures depression and one measures anxiety. The scores for of each scale can be between 0 and 21. Scores ranging from 8 to 10 are considered mild, from 11 to 14 moderate, and 15 to 21 severe [18]. For this study, 8 is used as the point indicating either anxiety or depression [16]. It is important to note that the scale shows symptoms of depression or anxiety in the last week and therefore does not necessarily indicate clinical depression.

#### 2.1.3. Pittsburgh Sleep Quality Index (PSQI)

The PSQI is a measurement of self-assessed sleep quality. High PSQI scores show low sleep quality, and low PSQI scores mean high sleep quality. Reliability of the PSQI is high, with a Cronbach alpha of 0.83. Seven components of sleep are evaluated by this instrument. These include sleep latency, sleep disturbances, sleep duration, sleep quality, sleep efficiency, use of sleep medications, and daytime dysfunction. The scores for each of these components are combined for a global score that can range from 0 to 21 [19]. An overall score from zero to five is normal sleep, while an overall score of greater than five is indicative of poor sleep quality [20].

#### 2.1.4. Relationship Assessment Scale (RAS)

The RAS [21] (Portuguese experimental version from Mesquita, Barbosa, and Figueiredo-Braga, 2014) is a 7-item instrument, with a five-point scale that measures general satisfaction with the marital or intimate relationship.

#### 2.1.5. Disease Activity Score 28 (DAS28)

The Portuguese version of the DAS 28 [22] was used to measure disease activity in RA patients. This index is calculated through joint examination, blood tests of inflammation, and global pain assessment. The lower the DAS28 score, the better the controlled disease [23].

#### 2.1.6. Pain Scale

Pain was self-reported to the telephone interviewer using a numerical scale from 1 to 10.

### 2.2. Cytokine Analysis

ELISA kits were purchased from eBiosciences, and serum cytokine levels were measured according to the manufacturer's directions. Peripheral blood samples were always collected in the morning (9–11 a.m.) into plastic tubes containing K3EDTA as an anticoagulant for plasma determinations or a gel separator for serum assays (BD Vacutainer, Franklin Lakes, NJ). Blood was centrifuged at 2000*g* for 10 minutes. Serum and plasma were frozen at −70°C for further determinations. Levels of IL-6, IL-10, and TNF-alpha were measured using ELISA for each participant.

### 2.3. Statistical Analysis

Significant differences in the demographical, clinical, and psychological variables between the SLE subjects, healthy controls, RA subjects, and depressed control subjects were determined using the independent *t*-test, Fisher's exact chi-squared, Mann–Whitney *U*, Wilcoxon rank sum, or Welch's tests when considered appropriate. Fisher's exact chi-squared was used in place of the standard chi-squared test, which would typically be utilized, due to the smaller sample size of the groups compared. The statistical tests used for the comparisons are indicated in the table legends.

Univariate analysis was accomplished by using generalized linear or logistic regression, using the Poisson function when appropriate, due to the Poisson distribution observed in the data. HADS depression scores were utilized as the dependent variable and with the individual variable suspected of showing a correlation to depression as the independent variable. Statistical analysis was performed using the statistical software R and SPSS (IBM). The multivariate analysis of the RA cohort was accomplished using logistic regression utilizing the Poisson function. HADS depression was used as the dependent variable. Only independent variables that were not co-related and had demonstrated a significant association in univariate analysis were used in the multivariate models to generate the best fit model. The best fit model was chosen based on the model demonstrating the lowest AICc value with the highest pseudo *R* ^2^ value.

To compare the rheumatoid arthritis patients that began biological therapy during the second month of the study, the appropriate paired *t*-test was utilized based on the data. For categorical variables, Fisher's exact test was used for the reasons previously described. An alpha value less than or equal to 0.05 was considered significant in all analyses.

### 2.4. Power Analysis

A post hoc power analysis was performed to test for the likelihood of type 2 errors, in which true correlations are missed due to inadequate sample size. The advantage of a post hoc power analysis is that it can consider the actual variation seen in the sample, as opposed to a projected variance, and thus provide a realistic analysis (Supplementary Table 1).

## 3. Results

Four different groups were recruited for this study: rheumatoid arthritis patients, lupus patients, primary depression patients, and healthy controls. The rheumatoid arthritis patients were significantly older than the other groups. They also had fewer years of education than the other groups. The RA patients were more likely to be married than the healthy controls, but less likely than the lupus patients. They were also less likely to be employed than the healthy controls, but not than the primary depression or lupus patients (Table 1). However, multivariate analysis revealed that these factors were not among the most significant contributors to depressive symptoms in rheumatoid arthritis. Univariate analysis was used to identify correlations between depression and clinical, treatment, and social factors (Tables 2 [Table tab3] [Table tab4] [Table tab5]–6). Multivariate analysis was used to identify the variables that most strongly predicted depression in the RA patients (Table 7).

Depressive symptoms were assessed for all participants using the HADS instrument. Therapeutics, clinical indicators, and psychological factors were all examined for correlation with symptoms of depression. Multiple correlations were found, encompassing all three areas. The use of the four different participant groups (rheumatoid arthritis, lupus (SLE), depressive disorder, and healthy controls) allowed the evaluation of the relative importance of each factor in rheumatoid arthritis. For example, if a particular condition correlated with depression in RA but not SLE, that would indicate that the condition was specific for RA and not a general feature of autoimmune arthritis.

### 3.1. Biological Therapeutics and Depression

In RA patients, the use of Tocilizumab, an IL-6 inhibitor, was associated with decreased depressive symptoms (*P* = 0.023, with an odds ratio of 0.70). No other biological agent was associated with depressive symptoms in either a positive or a negative manner. Interestingly, Sulfasalazine, a nonbiological treatment, was also associated with decreased depression (*p* = 0.012. OR = 0.58) (Table 2).

Fourteen RA patients were not initially on a biological therapeutic but were being treated with one at the time of the one-month follow-up. Of these patients, there was no significant change in depressive symptoms, although counter to what might be expected, they reported significantly higher fatigue (*p* = 0.01) in the follow-up month than in the first assessment. This fatigue was present even though there was no significant change in disease activity.

### 3.2. Cytokines and Depression

Levels of IL-6, IL-10, and TNF-alpha were measured using ELISA for each participant. IL-10 concentration correlated with depressive symptoms in patients with rheumatoid arthritis (*p* = 0.005, OR = 1.13), in that higher IL-10 levels correlated with more depressive symptoms. This correlation was also found in the healthy controls. Even though these individuals were not diagnosed with autoimmune disease or depression, IL-10 concentrations still correlated with depressive symptoms, although the association was not terribly strong (*p* = 0.047, OR = 1.05). However, in the patients with lupus, IL-10 levels were inversely correlated with depressive symptoms (*p* = 0.006, OR = 0.92), as has been seen for other diseases.

IL-6 and TNF-alpha correlations were also identified, although only in the primary depression patients. In these patients, IL-6 and TNF-alpha were both positively correlated with depressive symptoms (*p* = 0.011 and OR = 1.07 and *p* = 0.048 and OR = 1.00, resp.) (Table 3).

### 3.3. Clinical Values and Depression

Disease activity was strongly correlated with depression in the RA patients in this study. Both patient and physician assessments of overall disease activity strongly correlated with depressive symptoms (*p* < 0.001 for both measures) (Table 4). Clinical test results also correlated with depression in the RA patients. Sedimentation rate, C-reactive protein levels, and DAS28 scores all positively correlated with depressive symptoms (Table 4). In the lupus patients, these measures of inflammation did not correlate with depressive symptoms (Table 5).

Certain physical factors also correlated with depression. In both the RA patients and the lupus patients, body mass index correlated strongly with depressive symptoms (*p* < 0.001 and *p* = 0.003, resp.). BMI did not correlate with depressive symptoms in the patients with primary depression or in the healthy controls. Physical activity was strongly negatively correlated with depressive symptoms in rheumatoid arthritis (*p* < 0.001) and lupus (*p* = 0.047) (Tables 4 and 5). In fact, low physical activity is one of the factors identified by the multivariate analysis as being the most strongly predictive of depression using multivariate analysis (Table 7).

### 3.4. Pain, Psychosocial Measurements, and Depression

Self-reported pain scores correlated with depression in patients with RA (*p* < 0.001), lupus (*p* < 0.001), and primary depression (*p* < 0.001). Fatigue, relationship quality, and sleep quality were assessed for each study participant. All three of these measurements were strongly correlated with symptoms of depression in the RA patients (*p* < 0.001 for all). Therefore, depression in RA was correlated with worse overall sleep quality, poor relationship quality, and increased fatigue. Variants on this pattern were also observed for the other groups. Depression in lupus was correlated with fatigue (*p* < 0.001) and overall sleep quality (*p* = 0.012), but not marital relationship quality. In the depressive disorder patients, symptoms of depression were also associated with overall sleep quality (*p* = 0.008) and fatigue (*p* < 0.001). In the healthy controls, marital relationship quality was inversely associated with active depression (*p* = 0.009), and fatigue was directly associated (*p* = 0.005) (Table 6).

### 3.5. Multivariate Analysis

Multivariate analysis was performed to identify which of the variables was the most predictive for depressive symptoms and to ensure that differing sociodemographic factors between groups were not responsible for the outcomes of the study. This analysis showed that none of the sex, age, marital status, or years of education significantly predicted depressive symptoms. The best correlations with depressive symptoms were found for low physical activity (*p* = 0.003), poor relationship satisfaction as measured by the RAS (*p* < 0.001), and fatigue as measured by the FSS (*p* < 0.001) (Table 7).

## 4. Discussion

It is evident from these results that depression in rheumatoid arthritis is strongly linked to disease activity, as nearly every measure of systemic inflammation and disease activity was correlated with depressive symptoms. This is not the case in systemic lupus, where disease activity often bears little relation to depressive symptoms. Of the clinical indicators, only C-reactive protein levels were associated with active depression in lupus. This difference suggests that the root cause of depression is different in rheumatoid arthritis and lupus.

The results of the cytokine analysis support this finding. IL-10 is associated with depression in both lupus and rheumatoid arthritis, but whereas higher IL-10 levels correlate with depression in RA, lower levels correlate with depression in lupus. Since IL-10 is normally thought of as an anti-inflammatory cytokine, the association between IL-10 levels and depressive symptoms in RA is unexpected. It may be that increased IL-10 is serving as a proxy for increased inflammation. In this case, increased disease activity produces an increase in IL-10 as the normal immune regulatory systems attempt to damp down the inflammation. IL-10 seems to act differently in lupus and rheumatoid arthritis, being disease suppressive in RA and disease promoting in lupus [24], which may help to explain the contrasting results observed in this study.

IL-6 is also likely to be involved in depression. Treatment with the IL-6 inhibitor Tocilizumab was correlated with decreased depressive symptoms in the RA patients. Furthermore, IL-6 concentration correlated with active depression in the primary depression patients. This supports other studies that have suggested a role for IL-6 in depression [25, 26].

The relatively small number of patients in the control groups and the demographic differences between groups are the principal limitation of the present study. Although the longitudinal design of the study could be a strength, the short time between evaluations represents another limitation.

The use of biological therapeutics is a powerful tool not only for treatment but also for aid in the understanding of the mechanisms and pathogenesis of rheumatic diseases. The ability of these medications to inhibit cytokines or pathogenic cells provides a mechanism to examine the function of these factors in a very specific way, as was done with IL-6 in this work. Experiments are currently underway to examine the roles of cytokines in RA in an extended longitudinal analysis by examining patients on biologicals. These techniques are capable of being applied to any of the many diseases that are treated with biological therapeutics.

## Figures and Tables

**Table 1:** Sociodemographics of cohort.

*Characteristics*	All subjects	RA subjects	Healthy subjects		Depressed subjects		SLE subjects	
	(*N* = 209)	(*N* = 82)	(*N* = 22)	*p* value	(*N* = 32)	*p* value	(*N* = 73)	*p* value
Gender, number (%)								
Female	192 (92)	69 (84)	21 (95)	0.291	29 (91)	0.550	73 (100)	<0.001
Age, mean ± SD	49.27 ± 11.99	54.99 ± 10.08	43.14 ± 11.54	< 0.001	49.25 ± 14.03	0.040	44.70 ± 10.38	<0.001
Education (years), mean ± SD	8.43 ± 4.45	6.85 ± 3.99	13.55 ± 3.10	< 0.001	8.91 ± 4.31	0.017	8.88 ± 4.14	0.002
Education level, number (%)				< 0.001		0.200		<0.001
Primary (or less)	97 (46)	53 (65)	1 (5)		15 (47)		28 (38)	
Middle school	34 (16)	14 (17)	2 (9)		7 (22)		11 (15)	
High school	44 (21)	7 (9)	8 (36)		5 (16)		24 (33)	
College	32 (15)	7 (9)	10 (45)		5 (16)		10 (14)	
Graduate school	2 (1)	1 (1)	1 (5)		0 (0)		0 (0)	
Marriage status, number (%)				0.024		0.428		0.026
Unmarried	33 (16)	9 (11)	8 (36)		6 (19)		10 (14)	
Married	135 (65)	50 (61)	9 (41)		22 (69)		54 (74)	
Divorced	28 (13)	14 (17)	4 (18)		2 (6)		8 (11)	
Widowed	10 (5)	8 (10)	0 (0)		2 (6)		0 (0)	
Cohabiting	3 (1)	1 (1)	1 (5)		0 (0)		1 (1)	
Employment status, number (%)				0.002		0.137		0.452
Employed	88 (42)	28 (34)	16 (73)		16 (50)		28 (38)	

**Table 2:** Depressive symptoms correlated with medication in rheumatoid arthritis.

Medication	Pseudo *R* ^2^	AICc	Coefficient	Odds ratio (95% CI)	*p* value
Biological medications	0.154	572.98			
None			0.059	1.06 (0.83–1.37)	0.643
Abatacept			−0.143	0.87 (0.45–1.52)	0.642
Adalimumab			−0.143	0.87 (0.45–1.52)	0.642
Etanercept			0.011	1.01 (0.72–1.41)	0.948
Golimumab			0.011	1.01 (0.43–2.03)	0.978
Infliximab			0.113	1.12 (0.73–1.66)	0.588
Rituximab			0.145	1.16 (0.85–1.57)	0.361
Tocilizumab			−0.351	0.70 (0.52–0.95)	0.023
Classic medications	0.829	478.81			
Leflunomide			−0.284	0.75 (0.56–1.01)	0.061
Methotrexate			−0.207	0.81 (0.62–1.06)	0.125
Leflunomide and Methotrexate			0.301	1.35 (0.82–2.12)	0.209
Sulfasalazine			−0.546	0.58 (0.37–0.87)	0.012
Hydroxychloroquine + Leflunomide + Methotrexate			−0.441	0.64 (0.31–1.17)	0.184
Hydroxychloroquine + Methotrexate			0.090	1.09 (0.73–1.60)	0.650
Cyclosporine			0.029	1.03 (0.46–1.98)	0.937
Hydroxychloroquine + Leflunomide			−0.258	0.77 (0.30–1.61)	0.539
Sulfasalazine + Methotrexate			0.147	1.16 (0.70–1.86)	0.565
Hydroxychloroquine			0.147	1.16 (0.68–1.86)	0.565
Sulfasalazine + Hydroxychloroquine			0.147	1.16 (0.68–1.86)	0.565
Sulfasalazine + Leflunomide			−1.357	0.26 (0.04–0.81)	0.057

**Table 3 tab3:** Correlations between depressive symptoms and serum cytokine levels.

		Pseudo *R* ^2^	AICc	Coefficient	Odds ratio (95% CI)	*p* value
Rheumatoid arthritis	(*n* = 82)					
IL-6 (pg/mL), mean ± SD	44.04 ± 104.39	1.000	73.80	0.002	1.00 (1.00-1.00)	0.132
IL-10 (pg/mL), mean ± SD	4.12 ± 2.97	1.000	68.99	0.121	1.13 (1.03–1.22)	0.005
TNF-alpha (pg/mL), mean ± SD	109.92 ± 151.85	1.000	75.19	0.001	1.00 (1.00-1.00)	0.430
Lupus	(*n* = 73)					
IL-6 (pg/mL), mean ± SD	2.81 ± 2.18	1.000	83.69	0.055	1.06 (0.96–1.15)	0.229
IL-10 (pg/mL), mean ± SD	6.76 ± 4.06	1.000	76.47	−0.088	0.92 (0.86–0.97)	0.006
TNF-alpha (pg/mL), mean ± SD	60.66 ± 102.38	1.000	83.56	−0.001	1.00 (1.00-1.00)	0.243
Depressive disorder	(*n* = 32)					
IL-6 (pg/mL), mean ± SD	1.30 ± 2.23	0.970	143.99	0.070	1.07 (1.01–1.13)	0.011
IL-10 (pg/mL), mean ± SD	9.18 ± 15.66	0.962	146.13	0.008	1.01 (1.00-1.02)	0.059
TNF-alpha (pg/mL), mean ± SD	38.43 ± 67.75	0.962	146.01	0.002	1.00 (1.00-1.00)	0.048
Healthy controls	(*n* = 22)					
IL-6 (pg/mL), mean ± SD	1.11 ± 1.34	0.653	102.02	0.014	1.01 (0.84–1.18)	0.869
IL-10 (pg/mL), mean ± SD	3.07 ± 3.78	0.712	98.70	0.047	1.05 (1.00-1.09)	0.047
TNF-alpha (pg/mL), mean ± SD	33.77 ± 53.08	0.706	99.05	−0.005	1.00 (0.99–1.00)	0.109

**Table 4 tab4:** Correlations between depressive symptoms and clinical assessments in RA subjects.

	(*n* = 82)	Pseudo *R* ^2^	AICc	Coefficient	Odds ratio (95% CI)	*p* value
*Characteristics*						
Disease duration (years), mean ± SD	17.56 ± 11.48	0.252	461.64	−0.040	0.96 (0.88–1.05)	0.401
BMI, mean ± SD	26.46 ± 5.49	0.144	557.56	0.028	1.03 (1.01–1.04)	< 0.001
Pain score, median	6	0.411	527.31	0.114	1.12 (1.08–1.16)	< 0.001
Smoking, number (%)	0 (0)	< 0.001	570.11	−0.029	0.97 (0.77–1.22)	0.806
Drinking, number (%)	13 (16)	0.014	569.01	−0.129	0.88 (0.69–1.11)	0.290
Physical activity, number (%)	13 (16)	0.256	546.22	−0.668	0.51 (0.38–0.68)	< 0.001
Biologic medication, number (%)	42 (51)	0.027	570.09	−0.082	0.92 (0.73–1.18)	0.503
Classic medication, number (%)	55 (67)	0.030	569.89	−0.062	0.94 (0.75–1.19)	0.601
Disease activity (patient), mean ± SD	54.04 ± 30.16	0.880	426.21	0.002	1.01 (1.01–1.02)	< 0.001
Disease activity (doctor), mean ± SD	35.44 ± 27.03	0.890	419.81	0.013	1.01 (1.01–1.02)	< 0.001
*Laboratory*						
Sedementation velocity, mean ± SD	26.59 ± 22.27	0.834	448.13	0.010	1.01 (1.01–1.01)	< 0.001
C-reactive protein, mean ± SD	11.11 ± 17.43	0.778	468.09	0.006	1.01 (1.00-1.01)	0.007
DAS28, mean ± SD	5.04 ± 5.65	0.830	449.81	0.038	1.04 (1.02–1.05)	< 0.001
Brain leukocytes, mean ± SD	8.09 ± 3.40	0.707	484.34	0.010	1.01 (0.98–1.04)	0.425
Brain lymphocytes, mean ± SD	27.10 ± 9.84	0.713	482.76	−0.007	0.99 (0.98–1.00)	0.139
Anti-dsDNA, mean ± SD	10.66 ± 5.30	0.999	248.28	0.017	1.02 (1.00-1.04)	0.069

**Table 5 tab5:** Correlations between depressive symptoms and SLE clinical assessments.

	SLE subjects					
	(*n* = 73)	Pseudo *R* ^2^	AICc	Coefficient	Odds ratio (95% CI)	*p* value
*Characteristics*						
Disease duration (years), mean ± SD	17.81 ± 8.85	0.021	467.95	0.006	1.01 (1.00-1.02)	0.211
BMI, mean ± SD	24.51 ± 5.60	0.103	461.59	0.021	1.02 (1.01–1.03)	0.003
Smoking, number (%)						
Yes	13 (18)	0.032	467.14	−0.186	0.83 (0.65–1.05)	0.133
Drinking, number (%)						
Yes	7 (10)	0.006	469.03	0.100	1.11 (0.82–1.45)	0.489
Physical activity, number (%)						
Yes	24 (33)	0.054	465.43	−0.196	0.82 (0.68–0.99)	0.047
*Laboratory*						
Sedimentation velocity, mean ± SD	23.48 ± 20.96	0.355	440.12	0.003	1.00 (1.00-1.01)	0.220
C-reactive protein, mean ± SD	0.44 ± 0.57	0.191	454.67	0.120	1.13 (0.97–1.29)	0.094
Leukocytes, mean ± SD	6.73 ± 2.13	0.160	457.26	−0.003	1.00 (0.96–1.04)	0.884
Brain leukocytes, mean ± SD	6.22 ± 2.20	1.000	91.44	0.121	1.13 (1.04–1.23)	0.005
Lymphocytes, mean ± SD	28.89 ± 8.09	0.160	457.27	0.001	1.00 (0.99–1.01)	0.929
Brain lymphocytes, mean ± SD	29.05 ± 6.77	1.000	96.64	−0.022	0.98 (0.95–1.01)	0.117
Anti-dsDNA, mean ± SD	98.79 ± 111.01	0.829	370.64	< 0.001	1.00 (1.00-1.00)	0.990
Brain anti-dsDNA, mean ± SD	635.54 ± 1504.14	1.000	96.28	< 0.001	1.00 (1.00-1.00)	0.081

**Table 6:** Correlations between depression, pain, and psychosocial values.

		Pseudo *R* ^2^	AICc	Coefficient	Odds ratio (95% CI)	*p* value
Rheumatoid arthritis	(*n* = 82)					
Pain score, median	6	0.411	527.31	0.114	1.12 (1.08–1.16)	<0.001
PSQI, mean ± SD	8.09 ± 4.29	0.442	523.54	0.065	1.07 (1.05–1.09)	<0.001
Relationship Assessment Scale, mean ± SD	28.74 ± 9.54	0.989	328.73	−0.041	0.96 (0.95–0.97)	<0.001
Fatigue Severity Scale, mean ± SD	4.19 ± 1.57	0.627	491.36	0.261	1.30 (1.22–1.38)	<0.001
Lupus (SLE)	(*n* = 73)					
Pain score, median	5	0.328	440.46	0.078	1.08 (1.05–1.11)	<0.001
PSQI, mean ± SD	8.99 ± 2.97	0.081	463.29	0.037	1.04 (1.01–1.07)	0.012
Relationship Assessment Scale, mean ± SD	25.45 ± 4.19	0.818	370.67	0.014	1.01 (0.99–1.04)	0.252
Fatigue Severity Scale, mean ± SD	4.26 ± 1.38	0.551	410.99	0.268	1.31 (1.22–1.41)	<0.001
Depressive disorder	(*n* = 32)					
Pain score, median	5.5	0.600	185.10	0.132	1.14 (1.09–1.20)	<0.001
PSQI, mean ± SD	8.48 ± 2.61	0.782	173.46	0.067	1.07 (1.02–1.12)	0.008
Relationship Assessment Scale, mean ± SD	22.52 ± 7.21	0.983	129.17	0.012	1.01 (0.99–1.03)	0.250
Fatigue Severity Scale, mean ± SD	3.83 ± 1.49	0.876	158.17	0.216	1.24 (1.13–1.36)	<0.001
Healthy controls	(*n* = 22)					
Pain score, median	0	0.331	112.07	−17.810	0 (0–0)	0.993
PSQI, mean ± SD	5.18 ± 2.36	0.136	117.70	0.079	1.08 (0.99–1.18)	0.072
Relationship Assessment Scale, mean ± SD	28.06 ± 4.46	0.917	81.43	−0.066	0.94 (0.89–0.98)	0.009
Fatigue Severity Scale, mean ± SD	2.78 ± 1.05	0.311	112.71	0.291	1.39 (1.10–1.65)	0.005

**Table 7:** Multivariate Model for RA subject cohort.

Variables	Coefficient	Odds ratio (95% CI)	*p* value
Sex	0.164	1.18 (0.81–1.75)	0.401
Age (years)	−0.001	1.00 (0.98–1.02)	0.970
Education (years)	−0.071	0.93 (0.82–1.05)	0.273
Marital status	−0.016	0.98 (0.82–1.16)	0.854
Physical activity	−0.808	0.45 (0.25–0.74)	0.003
Relationship Assessment Scale	−0.041	0.96 (0.94–0.98)	< 0.001
Fatigue Severity Scale	0.206	1.23 (1.10–1.37)	< 0.001
Model fit summary	Pseudo *R* ^2^		*AICc*
	0.999		247.280

## Data Availability

All data used in the generation of this article are available upon request.

## Supplementary Materials

Supplementary 1 The post hoc power analysis of this project is included as Supplementary Table 1. This shows the likelihood of a type 2 statistical error for each comparison, based on the actual variance observed in our study.

Supplementary 2 The complete database that the study is based on is also included as Supplementary Table 2. This includes the complete data set used for each analysis.
